# An empirical exploration of female child marriage determinants in Indonesia

**DOI:** 10.1186/s12889-018-5313-0

**Published:** 2018-03-27

**Authors:** Lauren Rumble, Amber Peterman, Nadira Irdiana, Margaret Triyana, Emilie Minnick

**Affiliations:** 1 UNICEF Indonesia, Wisma Metropolitan II, 10-11th Floors, Jl. Jenderal Sudirman Kav.31, Jakarta, 12920 Indonesia; 2 UNICEF Office of Research—Innocenti, Piazza SS. Annunziata 12, 50122 Florence, Italy; 3 Plan International Indonesia, Menara Duta Building, Jl. H.R Rasuna Said Kav. B-9, Kuningan, Daerah Khusus Ibukota Jakarta, Kuningan, Indonesia; 4 Keough School of Global Affairs, University of Notre Dame, 3169 Jenkins Nanovic Halls, Notre Dame, 46556 United States

**Keywords:** Child marriage, Adolescent transitions, Structural determinants, Indonesia

## Abstract

**Background:**

Child marriage, defined as marriage before age 18, is associated with adverse human capital outcomes. The child marriage burden remains high among female adolescents in Indonesia, despite increasing socioeconomic development. Research on child marriage in Southeast Asia is scarce. No nationally representative studies thus far have examined determinants of child marriage in Indonesia through multivariate regression modeling.

**Methods:**

We used data from the nationally representative 2012 Indonesian Demographic and Health Survey and the Adolescent Reproductive Health Survey to estimate determinants of child marriage and marital preferences. We ran multivariate models to estimate the association between demographic and socioeconomic characteristics and the following early marriage outcomes: 1) ever been married or cohabited, 2) married or cohabited before 18 years, 3) married or cohabited before 16 years, 4) self-reported marital-age preferences and 5) attitudes approving female child marriage.

**Results:**

Among the child marriage research sample (*n* = 6578, females aged 20–24 at time of survey), approximately 17% and 6% report being married before 18 and 16 years old respectively. Among the marital preferences research sample (*n* = 8779, unmarried females 15–24), the average respondent preferred marriage at approximately 26 years and 5% had attitudes approving child marriage. Education, wealth and media exposure have protective effects across marriage outcomes, while rural residence is a risk factor for the same. There are significant variations by region, indicating roles of religious, ethnic and other geographically diverse factors.

**Conclusion:**

This research fills a gap in understanding of child marriage determinants in Indonesia. There appears to be little support for child marriage among girls and young women, indicating an entry point for structural interventions that would lead to lasting change. Future research efforts should prioritize rigorous testing of gender-transformative education and economic strengthening interventions, including cost-effectiveness considerations to better understand how interventions and policies can be leveraged to deliver on ending child marriage in Indonesia and globally.

## Background

Child marriage is a significant health and children’s rights concern in many low- and middle- income countries (LMICs). Globally, one in six adolescent girls between the ages of 15 and 19 years is married or in union, and as many as 700 million women were married as child brides in 2014 [1]. Child marriage, also known as early or forced marriage, is defined as any marriage in which either of the partners is under 18 years of age, with or without consent [2]. It significantly alters not only the lives of these girls themselves but also the life trajectories of their children. Global evidence shows, for example, that child marriage exposes girls to higher risk of maternal mortality. Complications during childbirth and pregnancy are one of the leading causes of death amongst adolescent girls [3, 4]. Children born to young mothers are also more likely to have poor nutritional and other health outcomes [5]. A study conducted in five LMICs found that children whose mothers were 19 or younger when they were born have a 20% to 30% higher risk of preterm birth and low birth weight [6, 7]. Married girls are also at greater risk of dropping out of school [8] and may face an increased risk of intimate partner violence [9, 10].

In September 2015, the General Assembly of the United Nations committed to targets eliminating all practices causing specific harm to women and girls, including child marriage (Target 5.3, Goal 5 of the UN Sustainable Development Goals - SDGs) [11]. To achieve this target by 2030, governments will require rigorous scientific data on the prevalence of child marriage in their countries and its structural determinants, to inform meaningful investment in program and policy responses.

However, most countries, especially LMICs, have very little data on the prevalence of child marriage or of other traditional practices harmful to girls [12]. Even less information is available about their (context-specific) structural and behavioral determinants, although some evidence is emerging. Child marriage appears to be more common in LMICs in comparison to high-income countries [13]. Unequal gender norms may drive child marriage. For example, some researchers have found that countries and societies with high gender inequality (e.g. laws and customs that exclude girls from decision-making or economic and political rights) are more likely to feature high prevalence of child marriage [14]. Education is commonly found to be a protective factor, both globally as well as in in studies from South Asia, with child brides consistently having lower education levels than women married over the age of 18 [15, 16]. Minimum marriage age laws have been shown to protect against child marriage [17]. Finally, poverty and rural residence are found to increase the risk of child marriage in every region of the world [18]. However, it should be noted that most research to date on determinants of child marriage is associational and thus fails to establish a causal link between background factors and adverse child marriage outcomes.

Most empirical research on child marriage has focused on South Asian and African countries where a high percentage of females marry before age 18. In contrast, relatively little research on the topic has been conducted in Southeast Asia. In Indonesia, most studies are limited to a specific geographic area and little nationally representative data or analysis is available. What data exists point to a significant problem: an estimated 17% of Indonesian girls are married before the age of 18, according to the most recent national Demographic Health Survey (DHS) conducted in 2012 [19]. According to UNICEF, Indonesia ranks approximately in the middle for countries with available data on marriage before the age of 18 in East Asia and Pacific region, with Laos and Solomon Islands ranking the highest at 37% and 28.3%, respectively and Mongolia and Vietnam ranking the lowest at 6.2% and 12.3% respectively [20]. However, due to the large population, Indonesia has one of the highest burdens of child marriage in the region and contributes substantially to the overall global burden of child brides [20, 21]. Although trends are promising, with median age at first marriage increasing among ever-married women age 25 to 49 (from an estimated 17.7 years at first marriage in 1991 to 20.1 in 2012), levels are still unacceptably high. A 2016 report by the National Statistics Bureau and UNICEF Indonesia finds that using bivariate analysis, child marriage is associated with rural residence, poorer housing conditions and households with lower levels of expenditure; all categories associated with poverty [21]. However, insufficient analysis is available to explain the wide variance in child marriage rates across the country, including within districts and provinces.

Indonesia, with more than 255 million people, is home to the world’s largest Muslim population. Geographically and culturally diverse, the country has emerged as a significant economic and political power. While still a LMIC, annual gross domestic product (GDP) growth has averaged almost 6% in recent years [22]. Despite these advances, children in Indonesia face a number of serious challenges. Some studies claim that as many as half of Indonesian children live in poverty [23]. Under-five mortality is showing gradual improvement and is currently at 40 deaths per 1000 live births, although some eastern provinces show much higher rates [19]. Stunting amongst children below five years remains high, at roughly 37% [24]. Maternal mortality is at 359 deaths per 100,000 live births and has been on the increase [19].

The United Nations Committee on the Convention on the Rights of the Child (UNCRC) has urged the Indonesian government to take urgent action to implement stronger protections for girls against all forms of violence, including child marriage [25]. Although Indonesia ratified the UNCRC in 1990, its laws protecting children from marriage are inconsistent [26]. For example, the 2002 Child Protection Law prohibits any child from getting married before 18 years, but the minimum age for marriage (with parental consent) is 16 for girls and 19 for boys by Article 7 of the 1974 Marriage Law. The Marriage Law also provides opportunities for dispensation, allowing parents to marry their children legally at a younger age, even without their expressed consent. Families may also choose to adhere to cultural law (*adat*) in Indonesia, in which ideas of minimum age of marriage and consent differ widely with the various *adat* systems and regions across the country. Traditional attitudes about gender and women’s role in society can also influence child marriage. One study from 2015 finds that parents and the community may arrange female marriage as a remedy for rape [27].

In this paper, we analyze nationally representative data from Indonesia to examine structural factors predicting child marriage dynamics among a sample of women aged 20 to 24 to inform policy and programs. Understanding determinants of child marriage in Indonesia is of high relevance to the global understanding of child marriage dynamics as Indonesia contributes significantly to the regional and global burden of child marriage. Indonesia is perceived as a high-performing economy, however there are still significant challenges for women and children that prevent sustainable development for all its citizens. A multidimensional approach to understanding, and addressing, child marriage is needed. The analysis contributes to the literature in two main ways. First, to our knowledge, this is the first analysis of determinants of child marriage using a multivariate analysis of nationally representative, large-scale data. As Indonesia is a diverse nation, it is important from a policy perspective to situate the findings of smaller scale and regionally specific findings in a national context. Second, empirical analysis of marital preferences and attitudes are virtually nonexistent, but are thought to perpetuate child marriage dynamics at the societal level. Therefore, it is useful to understand what observable underlying factors are associated with preferences and harmful attitudes among unmarried young females. We conclude with a discussion of policy and program opportunities to reduce rates of child marriage as well as reflections on a research agenda to inform policy and programming efforts to end child marriage.

## Methods

The data for this analysis come from two related sources. The main analysis, on prevalence and structural determinants of child marriage, uses data from the 2012 Indonesia DHS, implemented by Statistics Indonesia (*Badan Pusat Statistik*, BPS) in collaboration with the National Population and Family Planning Board and the national Ministry of Health, with technical assistance from ICF International [19]. The DHS provides nationally representative data primarily from females of reproductive age (15–49) and are typically implemented approximately every five years in LMICs to monitor and understand population health and demographics. Further information, including information on sampling and questionnaire design, is available on the DHS website (http://www.dhsprogram.com/). As a special addition to the DHS 2012, a complementary data collection, the Adolescent Reproductive Health (ARH) survey was conducted, which sampled never-married women and men aged 15–24 and covered topics including marital aspirations and knowledge and risk behavior regarding sexual activities and HIV, among others [28].

Following international definitions [29], for the main analysis we focused on indicators of the following child marriage outcomes: (1) ever married or cohabited, (2) married or cohabited before the age of 18, and (3) married or cohabited before the age of 16.^1^ The DHS questions eliciting information on these items are presented in Table 1. Similarly, per international standards on child marriage statistics, we limited the DHS analysis to a sample of women aged 20 to 24, as these women have passed through the full age range(s) for classification of child marriage. There is also some evidence suggesting that women in older age cohorts have higher disclosure rates for early transition indicators as compared to younger women in the 15–19 age range [30]. We supplemented the child marriage analysis using the full ARH sample to analyze the determinants of marital-age preferences and attitudes approving child marriage among unmarried female respondents aged between 15 and 24. We focused on two questions, relating to self-reported preferences on the future age of marriage of the respondent and attitudes around the best age of marriage for females in general (Table 1). In our construction, attitudes are defined as an individual’s favorable or unfavorable disposition towards an object or practice, independent of what may be deemed appropriate in a particular social context [31].^2^ Note that since the DHS and ARH samples originate from different sampling frames (the latter being never married youth), they are not comparable or linked in our analysis in any way.

**Table 1:** Definitions of early marriage outcome indicators among females from 2012 Demographic and Health Survey and 2012 Adolescent Reproductive Health Survey

Indicators	Survey	Age range (years)	Survey question(s) details
(1) Ever married or cohabited	DHS	20–24	Question 1*: Are you currently married or living together with a man as if married?* Possible responses include: (1) *Yes, currently married*, (2) *Yes, living with a man*, and (3) *No, not in a union*.
			Question 2: *Have you ever been married or lived together with a man as if married?* Possible responses: (1) *Yes, formerly married*, (2) *Yes, lived with a man*, and (3) *No*.
(2) Married or cohabited age < 18 years	DHS	20–24	Question 1 (following ever marriage or cohabitation classification)*: How old were you when you first started living with him?* Responses are given in the form of the respondent’s age in years (at the time of cohabitation or marriage).
(3) Married or cohabited age < 16 years	DHS	20–24	
(4) Martial-age preferences (years)	ARH	15–24	Question 1: *At what age would you like to be married?* Responses are given in the form of the respondent’s preferred age at marriage in years.
(5) Attitudes approving child marriage (< 18 years)	ARH	15–24	Question 1: *In your opinion, what is the best age for a woman to get married?* Responses are given in the form of the respondent’s opinion of the best age at marriage in years for females.

We conducted a multivariate probit regression analysis to explore structural determinants of child marriage outcomes. A probit model is a standard regression when using binary indicators (taking the value of 0 or 1) to estimate the probability that an observation falls into a specific category [32]. The probit model is similar to a logistic regression model, with some advantages in terms of interpretation as coefficients can be expressed as marginal effects and interpreted as percentage point (pp) changes with respect to the outcome of interest. We assume a standard model:

1 $$ \Pr \left(Y=1|X\right)=\phi \left({X}^T\beta \right), $$

Where Pr is the probability (e.g. of child marriage) and ɸ is the cumulative distribution function of the standard normal distribution. The parameters (ß) are estimated using maximum likelihood. Operationally, if we assume Y* is a latent variable which we observe when Y* > 0 (= 1), and otherwise as equal to zero, then we can estimate the following model:

2 $$ {\mathrm{Y}}^{\ast }=X\upbeta +\upvarepsilon, $$

where the error term is normally distributed, ε ~ N (0, σ ^2^),

We select a vector of independent variables (β) based on available underlying structural characteristics hypothesized to be linked to early marriage. Focusing on structural determinants mitigates against the possibility they are behavioral choice factors or likely to be reversely casually linked to early marriage [33]. Independent variables include certain individual characteristics (*age indicators in years*; *education attainment in years*; *number of siblings in childhood household*; *exposure to media including radio*, *newspaper, and television*), household characteristics (*wealth quintiles*), and place of residence (*urban/rural*). Household wealth quintiles were pre-computed in using principal component analysis and including household-level durable asset ownership and housing quality indicators (e.g. floor, wall and roof type, access to water and sanitation). In all analyses, we adjusted for province fixed effects (across 33 provinces,^3^ with base category Jakarta), however suppressed coefficients for provinces in Table output due to the large number and instead reported a joint test of significance. Province fixed effects may absorb time-invariant unobserved heterogeneity related to, for instance, ethnic and religious diversity as well as differences in the level of development between provinces. Coefficients from probit models are reported as marginal effects (Tables 3 and 5). All analyses accounted for the complex survey design and sampling weight, with standard errors clustered at the primary sampling unit (PSU) level.

There are two differences to note between the DHS and ARH analysis. First, as the preferred age at marriage outcome is continuous, we utilized an ordinary least squares (OLS) regression approach, and second, as the number of siblings is not available in the ARH data, we omitted this determinant from the analysis. Finally, for all other models, to understand if our results are sensitive to our choice of probit specification, we replicated our analysis using linear probability models and found qualitatively similar results (available upon request). The study is exempt from ethical review, as our method involved conducting secondary analysis of publicly available and de-identified data.

## Results

### Descriptive statistics

Table 2 shows weighted descriptive statistics for our child marriage sample, of 6578 females ages 20–24 (Column A) and the statistics for the marital preferences and attitudes sample of 8779 unmarried females age 15–24 (Column B). As the majority of indicators are binary, we can interpret means (proportions) as percentages of the sample with each particular outcome or background characteristic. In the DHS sample, approximately 62% of females have ever been married or cohabited, 17% before the age of 18, and 6% before the age of 16. Further disaggregation of the sample shows that over 95% of those who reported ever being married are still currently married, while 3% reported being divorced or separated, 1% reported cohabiting or being a widow with 1% not reported. The age distribution of the sample is split roughly evenly by years of age (average age is 22 years). Most respondents had partial or completed secondary education (56%); fewer had only elementary education (20%) or at least some post-secondary or higher (22%). There were varying levels of exposure to media, with approximately 86% of respondents reporting they watch TV at least once a week, and fewer reports of listening to the radio (23%) and reading a newspaper or magazine (15%). Approximately 47% of the sample resides in a rural area.

**Table 2:** Summary statistics for child marriage outcome and background characteristics among females age 20–24 years, from the 2012 Indonesian Demographic and Health Survey among females aged 15–24, 2012 Indonesian Adolescent Reproductive Health Survey

	DHS (2012)		ARH (2012)	
*Outcomes*	Mean (proportion)	Standard error	Mean (proportion)	Standard error
Ever married or cohabited	0.617	(0.014)	n/a	n/a
Married or cohabited age < 18 years	0.170	(0.008)	n/a	n/a
Married or cohabited age < 16 years	0.055	(0.005)	n/a	n/a
Martial-age preferences (years)	n/a	n/a	25.576	(0.039)
Attitudes approving child marriage (< 18 years)	n/a	n/a	0.053	(0.004)
*Background characteristics*				
*Age splines (DHS/ARH samples)*				
Age = 20 /15 years	0.198	(0.007)	0.116	(0.005)
Age = 21 /16 years	0.195	(0.007)	0.137	(0.005)
Age = 22 /17 years	0.201	(0.008)	0.130	(0.005)
Age = 23 /18 years	0.211	(0.008)	0.114	(0.005)
Age = 24 /19 years	0.194	(0.008)	0.107	(0.005)
Age = * /20 years	n/a	n/a	0.100	(0.004)
Age = * /> 20 years	n/a	n/a	0.296	(0.007)
*Education*				
No education	0.014	(0.002)	0.002	(0.001)
Some or complete primary	0.204	(0.010)	0.115	(0.008)
Some or complete secondary	0.563	(0.012)	0.499	(0.009)
Secondary	0.219	(0.012)	0.384	(0.010)
Total number of siblings	3.385	(0.053)	n/a	n/a
*Household wealth*				
Wealth quintile 1	0.172	(0.009)	0.146	(0.008)
Wealth quintile 2	0.197	(0.010)	0.196	(0.008)
Wealth quintile 3	0.213	(0.010)	0.224	(0.008)
Wealth quintile 4	0.213	(0.010)	0.211	(0.009)
Wealth quintile 5	0.205	(0.012)	0.224	(0.011)
*Exposure to media*				
Listens to radio at least once a week	0.229	(0.008)	0.311	(0.009)
Reads newspaper or magazine at least once a week	0.150	(0.001)	0.206	(0.009)
Watches TV at least once a week	0.864	(0.007)	0.865	(0.007)
Rural	0.465	(0.019)	0.421	(0.018)
Sample size	6578		8779	

Notes: Weighted by primary sampling unit for national representativeness. All indicators with the exception of marital-age preferences and total number of siblings are binary variables and expressed as a proportion of the sample ranging from 0 to 1. Wealth quintiles are pre-computed using principle component analysis and a range of household-level durable asset ownership and housing quality indicators

For the ARH sample, the average preferred age of marriage is 25.6 years (ranging from age 17 to age 50).^4^ Approximately 5% of sample reported that in general, females should marry before the age of 18, indicating a relatively small percentage of the sample reported attitudes supporting child marriage. Most women had at least some secondary education (50% had some or completed secondary education, 38.4% had more than secondary education), 11.5% had some or completed primary education, and less than 1% had no education. Similar to the DHS sample, there were varying levels of exposure to media among unmarried females, with approximately 86% of females reporting they watch TV at least once a week, 31% listening to the radio, and 20% reading a newspaper or magazine. Approximately 42% of the sample resides in a rural area.

**Fig. 1:**
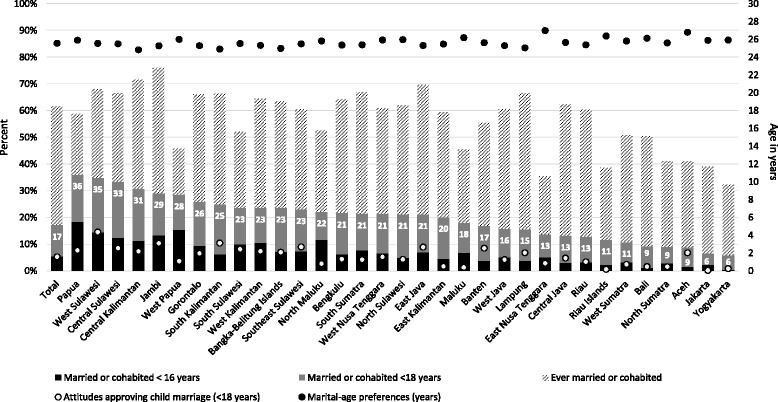
Marriage outcomes by province among females aged 20–24 (2012 Indonesian Demographic and Health Survey) and marital preferences and attitudes among females aged 15–24 (2012 Indonesian Adolescent Reproductive Health Survey). Notes: Means are weighted by primary sampling unit for national representativeness and ranked by prevalence of marriage or cohabitation < 18 years, from left to right

To help understand the variation in outcomes across Indonesia, we present a graphical depiction of indicators by province (Fig. 1). On the left most bar of Fig. 1, we present means for the full sample, followed by means for each province sorted from highest to lowest prevalence in terms of our lead indicator, child marriage (married before 18 years). The figure shows that prevalence of child marriage (solid gray bar) ranges from a high of 36% in Papua to a low of 6% in Yogyakarta. Eighteen regions have child marriage levels above 20% (or 1 in 5 females), with highest prevalence in Papua, West Sulawesi, Central Sulawesi and Central Kalimantan (all above 30%). Prevalence of marriage before the age of 16 (solid black bar) follow similar trends, with the highest being in Papua (18%), West Papua (15%), West Sulawesi (14%) and Jambi (13%). For many provinces, the prevalence of marriage before the age of 16 is low and nearly non-existent (Yogyakarta, Bali and Aceh, all under 2%). The prevalence of ever being married (shaded bar) range from 76% in Jambi to 35% in East Nusa Tenggara, and did not always follow the same trend as for the age-specific child marriage indicators. Moving to the attitudes supporting child marriage (white dot), provincial variation in reporting under 18 as a preferred age of marriage ranges from 15% in West Sulawesi to under 1% in Jakarta, Yogyakarta and Riau Islands. Finally, in contrast to some of the other indicators, there was relatively little variation in preferred age at marriage (black dot), fluctuating from 24 to 27 years. With the caveat that sample sizes were reduced when disaggregated by province, overall there appear to be important differences across geographic area in terms of outcomes.

### Determinants of child marriage

Table 3 shows the results of the probit multivariate regression models and marginal effects for structural determinants predicting child marriage outcomes. As expected, current age was a significant determinant of ever having been married or cohabited; however, this was less important for marriage or cohabitation before 18 or 16 years, where current age was generally insignificant. Across all outcomes, education was highly predictive and protective of early marriage outcomes (joint χ^2^ tests across education splines are significant in all models). For example, as compared to females with some or complete secondary education, those with lower levels (some or complete elementary) were at 6–12 percentage points (pp) increased risk of early marriage, while those who had at least some post-secondary education were at 9–33 pp decreased risk. Having more siblings when growing up reduced one’s risk of early marriage, which may indicate that there was increased pressure to marry or attention to marrying off children in smaller families. Wealth was consistently protective, with wealthier households decreasing risk of early marriage. This protective effect was generally increasing in magnitude with higher quintiles. For example, as compared to the poorest households (first quintile), females in quintiles 2 and 3 were 4 pp less likely to be married or cohabiting before 18 years, while females in quintiles 4 and 5 were 7 pp and 11 pp less likely to be married or cohabiting before 18 years, respectively (Table 3, Column B). Media exposure was generally protective, although it is likely that these measures are highly co-linear and joint significance is only achieved for ever married outcomes. In all models, rural residence was a risk factor: rural females were 2–11 pp more likely to ever be married or be married early (before age 16 and 18, respectively) as compared to urban counterparts. In all models, provincial fixed effects were jointly significant, indicating the importance of area of residence and of other regionally distributed structural determinants, such as religious and ethnic diversity.

**Table 3:** Probit models predicting determinants of child marriage dynamics among females aged 20–24, 2012 Indonesian Demographic and Health Survey

	Ever married or cohabited	Married or cohabited age < 18 years	Married or cohabited age < 16 years
*Age splines (years, base = age 20)*	(A)	(B)	(C)
Age 21 (years)	0.08	−0.01	0.01
	(0.01)***	(0.01)	(0.01)
Age 22 (years)	0.15	−0.00	0.01
	(0.01)***	(0.01)	(0.01)
Age 23 (years)	0.22	−0.01	0.00
	(0.01)***	(0.01)	(0.01)
Age 24 (years)	0.28	−0.02	0.00
	(0.02)***	(0.01)	(0.01)
*Education (base = some or complete secondary)*			
No education	−0.06	0.08	0.07
	(0.04)	(0.03)**	(0.02)***
Some or complete elementary education	0.06	0.12	0.07
	(0.02)***	(0.01)***	(0.01)***
> secondary education	−0.33	−0.25	−0.09
	(0.01)***	(0.02)***	(0.02)***
Number of siblings (logged)	−0.02	−0.01	− 0.01
	(0.01)***	(0.00)***	(0.00)**
*Wealth quintiles (base = quintile 1)*			
Wealth quintile 2	−0.06	−0.04	− 0.02
	(0.02)***	(0.01)***	(0.01)**
Wealth quintile 3	−0.11	−0.04	− 0.01
	(0.02)***	(0.01)***	(0.01)
Wealth quintile 4	−0.12	−0.07	− 0.04
	(0.02)***	(0.02)***	(0.01)***
Wealth quintile 5	−0.19	−0.11	− 0.04
	(0.02)***	(0.02)***	(0.01)***
*Media exposure indicators*			
Listens to radio at least once a week	−0.05	−0.01	− 0.01
	(0.01)***	(0.01)	(0.01)
Reads newspaper/magazine at least once a week	−0.03	−0.04	− 0.01
	(0.01)**	(0.02)**	(0.01)
Watches TV at least once a week	−0.00	− 0.00	− 0.00
	(0.01)	(0.01)	(0.01)
Rural *(base = urban)*	0.11	0.05	0.02
	(0.01)***	(0.01)***	(0.01)***
Adjusted for provincial fixed effects	Yes	Yes	Yes
χ^2^-stat provincial fixed effects (p-value)	116 (0.000)	93 (0.000)	66.8 (0.000)
χ^2^-stat education indicators (p-value)	653 (0.000)	335 (0.000)	174 (0.000)
χ^2^-stat media exposure indicators (p-value)	24.1 (0.000)	6.06 (0.109)	2.01 (0.570)
Sample size	6578	6578	6578
Pseudo-R^2^	0.29	0.19	0.19

Notes: Coefficients are from probit models reported as marginal effects and standard errors in parenthesis clustered at the primary-sampling unit (PSU) level. * *p* < 0.1 ** *p* < 0.05; *** *p* < 0.01. Wealth quintiles are pre-computed in the DHS using principle component analysis and a range of household-level durable asset ownership and housing quality indicators

To help contextualize the marriage outcomes of the married young women in our sample, in Table 4 we compared mean descriptive measures of our sample who reported having married or cohabited before age 18 (Column A) to those who reported having married or cohabited at or after age 18 (Column B). We reported weighted means for five proxy indicators of marriage equity and quality of marriage match: (1) average age at first marriage or cohabitation; (2) whether the age gap between partners is greater than 5 years; (3) a partner’s having no, or incomplete primary education; (4) a partner’s having been unemployed or not worked in the last 12 months and (5) a summary indicator of the number of joint or sole decisions a woman typically participates in across four decisions (woman’s own health, large household purchases, visits to family or relatives and what to do with husband’s/partner’s earnings). Women’s decision-making questions are frequently utilized in the literature as a proxy measure for female intra-household bargaining power, however the number and range of questions operationalized varies by survey [34, 35].

**Table 4:** Summary statistics for marriage outcomes among ever married or cohabiting females aged 20–24, 2012 Indonesian Demographic and Health Survey

		(A) Married or cohabited < 18 years		(B) Married or cohabited ≥18 years		p-value (Difference A = B)
*Outcomes*	Sample size	Mean (proportion)	Standard error	Mean (proportion)	Standard error	
Age at first marriage or cohabitation (years)	3888	15.82	(0.058)	19.86	(0.048)	*0.000*
Partner age differences > 5 years	3693	0.578	(0.025)	0.381	(0.015)	*0.000*
Partner has no or incomplete primary education (lowest category)	3888	0.160	(0.015)	0.063	(0.007)	*0.000*
Partner unemployed or not working (last 12 months)	3880	0.020	(0.021)	0.014	(0.014)	*0.666*
Female participation in household decisions (0–4)	3678	3.29	(0.051)	3.35	(0.030)	*0.462*

Notes: Data comes from the 2012 Indonesian Demographic and Health Survey and are weighted by primary sampling unit for national representativeness; p-value from unadjusted probit or ordinary least squares regression. Sample for partner age differences reduced due to trimming of highest and lowest 1% of distributions and due to missing data. All indicators with the exception of age at first marriage and female participation in household decisions are binary variables and expressed as a proportion of the sample ranging from 0 to 1. Female participation in household decisions is an indicator ranging from 0 to 4 indicating her participation either solely or jointly in four decisions: (1) woman’s health care decisions, (2) large household purchases, (3) visits to family or relatives and (4) what to do with money husband earns

The results of the mean difference tests (right most column, Table 4) showed that in three out of five cases, females who marry or cohabit before the age of 18 had more unequal marital outcomes as compared to their counterparts who marry or cohabit at or after age 18. In particular, 16% of early-married or cohabiting females had partners with no or incomplete primary education, as compared to 6% among females who marry or cohabit later. Particularly striking was the percentage of females in the former group with a greater-than-five-year age gap between partners (approximately 58%) versus that among females in the latter group (38%). However, there was no difference between the two groups in terms of partner being unemployed or not working and female decision-making power. This may be because the proportion of partners not working was very low on average (< 2%) and women indicated participating in a high number of decisions (3.3 on average out of 4). As marriage equity and quality is multi-dimensional and likely to vary by cultural setting, it was likely that better proxy measures would capture additional inequalities. In addition, these average differences should be taken as illustrative only, as we did not attempt to draw a causal relationship between the two groups of women. Despite this, overall the analysis showed that structural determinants were not only influencing the timing of marriage and cohabitation, but also indirectly contributing toward higher inequalities among partners in child marriages.

### Determinants of marital preferences and attitudes

Table 5 shows the results of OLS and probit models for young unmarried women’s marital age preference as well as attitudes supporting child marriage. As expected, age was a significant predictor of marital age preference, with older unmarried women being more likely to report a higher age preference (Column A). Higher education levels (particularly post-secondary education) were also associated with higher marriage age preferences. Wealth was generally not a significant predictor of marriage age preference, unlike its status for child marriage, with the exception of the highest wealth quintile, which was a protective factor (raising the preferred age of marriage by 0.19 years). Media exposure had an inconsistent association with marriage age preference, whereby exposure to newspapers and magazines was associated with higher marriage age preference (by 0.11 years), while other media have no significant effects. Finally, similar to child marriage, rural residence was associated with lower marriage age preference (by 0.21 years).

**Table 5:** Probit and ordinary least squares models predicting determinants of marital-age preferences and attitudes approving child marriage among unmarried females aged 15–24, 2012 Indonesian Adolescent Reproductive Health Survey

	Martial-age preferences (years)	Attitudes approving child marriage (< 18 years)
*Age splines (years, base = age 15)*	(A)	(B)
Age 16 (years)	0.17	−0.01
	(0.11)	(0.01)
Age 17 (years)	0.30	0.00
	(0.11)***	(0.01)
Age 18 (years)	0.22	0.01
	(0.12)*	(0.01)
Age 19 (years)	0.44	−0.01
	(0.12)***	(0.01)
Age 20 (years)	0.67	0.00
	(0.12)***	(0.01)
Age > 21 (years)	0.97	0.01
*Education (base = some or complete secondary)*	(0.10)***	(0.01)
No education	−0.27	0.02
	(0.44)	(0.03)
Some or complete elementary education	−0.79	0.03
	(0.10)***	(0.01)***
> secondary education	0.43	−0.03
	(0.07)***	(0.01)***
Wealth quintile 2 *(base = quintile 1)*	−0.04	−0.01
	(0.09)	(0.01)**
Wealth quintile 3	0.04	−0.02
	(0.09)	(0.01)***
Wealth quintile 4	0.14	−0.03
	(0.10)	(0.01)***
Wealth quintile 5	0.19	−0.04
	(0.10)**	(0.01)***
Listens to radio at least once a week	0.01	0.00
	(0.06)	(0.01)
Reads newspaper or magazine at least once a week	0.11	0.01
	(0.06)*	(0.01)
Watches TV at least once a week	−0.04	−0.00
	(0.08)	(0.01)
Rural *(base = urban)*	−0.21	0.02
	(0.06)***	(0.01)***
Adjusted for provincial fixed effects	Yes	Yes
χ^2^-stat provincial fixed effects (p-value)	7.45 (0.000)	117.50 (0.000)
χ^2^-stat education indicators (p-value)	56.22 (0.000)	88.70 (0.000)
χ^2^-stat media exposure indicators (*p*-value)	1.02 (0.384)	2.41 (0.492)
Sample size	8779	8779
R^2^ / Pseudo-R^2^	0.11	0.12

Notes: Coefficients for marital-age preferences models are from ordinary least squares regression models and coefficients for attitudes approving child marriage (< 18 years) are from probit models reported as marginal effects. Standard errors in parenthesis clustered at the primary-sampling unit (PSU) level. * p < 0.1 ** p < 0.05; *** p < 0.01. Wealth quintiles are pre-computed in the ARH using principle component analysis and a range of household-level durable asset ownership and housing quality indicators

Next, we explored the prevalence and determinants of harmful attitudes suggesting that the best age of marriage for women is less than 18 (child marriage), using probit models (Column B). Age was not found to be associated with attitudes around child marriage. However, women with lower education and women in rural areas were more likely to express the attitude that child marriage is acceptable. Household wealth was significantly protective and media exposure was not significant. As in the previous DHS analyses, in all models, provincial fixed effects were jointly significant, indicating the importance of area of residence and of other geographically distributed structural determinants.

## Discussion

This study adds to the growing evidence on the epidemiology of child marriage in Southeast Asia, and globally. Our findings confirm that a large proportion of females are still entering into child marriage and cohabitation situations in Indonesia, placing young mothers and their children at significant risk. We find that across outcomes, many of the same risk and protective factors are significant predictors of child marriage related outcomes. Like other studies, our findings suggest that in Indonesia, education is a strong protective factor against child marriage and against certain harmful marital preferences and attitudes. These findings indicate that, all else being equal, policies that promote girls’ completion of secondary schooling could lead to meaningful decreases in child marriage. The protective effects of urban residence and wealth confirm and build upon the findings from other studies from Indonesia [22, 36] and suggest that the government and partners make greater investments in social protection and poverty eradication. Poor households may see child marriage as economically beneficial in the short-term, but it does not improve the economic status of the household over the long-term or provide financial security for the future, potentially due to the lost financial capital of married girls and women not working [37]. Indeed, Indonesian women are less likely to have ever worked for pay and women work fewer hours than men [38]. From this perspective, child marriage in Indonesia likely maintains or exacerbates poverty, rather than alleviating it. Importantly, our findings indicate that nearly universally, unmarried females (aged 15 to 24) have attitudes rejecting child marriage and would prefer to enter into partnerships as adults. This is potentially indicative of a growing norm that the practice of child marriage should be stopped. Together, these findings provide compelling arguments in support of broader child marriage prevention efforts in Indonesia, including potential legislative reform of the Indonesian Marriage Law.

There are several limitations of this study primarily resulting from the observational nature of the analysis. As the data are cross-sectional, we were not able to track respondents over time to explore whether factors evident at earlier ages are causally linked to later marriage outcomes—or, similarly, if early marriage is causally linked to adverse later-life health or well-being outcomes. For example, while education for adolescent girls is a protective factor, and should continue to be promoted, the direction of the relationship between marriage and education needs to be further explored in order to accurately inform programming options. While it appears that females who receive more education are protected from child marriage, child marriage may on the other hand often become a reason to leave school. We find that the number of siblings a female had while growing up decreases the likelihood of child marriage, which is counterintuitive and merits further investigation as it may be an artifact of co-linearity with other omitted variables.^5^ In addition, there may be relevant risk factors we were not able to identify, for example religious and ethnic diversity or gender norms at the community level. Despite these limitations, the data are nationally representative and therefore have potential to inform more robust policy and programming recommendations based on simple analyses showing population-level dynamics.

## Conclusion

Child marriage has harmful life-long effects, both for the current and future generations. Like other studies from Indonesia, this analysis shows that similar structural factors are important in predicting both child marriage, as well as preferences and attitudes surrounding the practice. A recent systematic review including grey literature identified 11 high quality interventions and evaluations, six of which had some positive impact on reducing child marriage or increasing the age of marriage and can provide guidance in selecting which intervention design is likely to be successful in a given context such as Indonesia [39]. Our study suggests that the Government of Indonesia, private sector and civil society partners should prioritize secondary education for all girls and boys, and ensure that social protection financing is sufficient to reach poor households where vulnerable girls reside. The Indonesian Village Law provides an opportunity to leverage local government resources in this regard, by allocating a significant amount of funds for social services (up to 1 billion Indonesian Rupiah) per village. Strong civil society voices, and evidence around child marriage, including strategies to eliminate it, can influence these allocations. Specific opportunities for improving girls’ access to protective, including economic, assets should also be explored in line with good practice internationally [40]. In addition, the Government and other actors, including religious and traditional leaders, could initiate public awareness campaigns and mobilize community engagement to disseminate messages about children’s rights, gender inequality and the harms of child marriage. Based on our findings, these campaigns could build on the knowledge that a significant portion of the population that have attitudes and preferences against marriage before 18 years. Finally, as Indonesia is a highly diverse country with a range of religious, linguistic, and ethno cultural groups, the results here have highlighted the importance of investigating the specific factors that drive child marriage at the regional level, with extensive reference to dynamics such as religious affiliation.

Ending child marriage is a critical gender target of the SDGs and the global 2030 agenda. Already, Indonesia has embraced the SDG targets, reflecting commitments to the SDGs in its development planning and budgeting processes ahead of many of its neighboring countries. To achieve the 2030 agenda, however, policymakers must take bold action to place equity concerns and the rights and protection of girls in particular, at the heart of future development efforts. Future research efforts should prioritize rigorous testing of gender-transformative education and economic strengthening interventions, including cost-effectiveness considerations to better understand how interventions and policies can be leveraged to deliver on ending child marriage in Indonesia and globally.

## Footnotes

1 Following international definitions, we also estimated marriage before the age of 15, however due to low prevalence (2.7%), we were not able to estimate the full model with regional fixed effects among the full sample. However, the contribution of covariates among the key structural determinants in models excluding regional fixed effects was very similar in terms of magnitude and significance of coefficients using this definition of child marriage as compared to the model presented in the manuscript.

2 Attitudes are similar but distinct from measures of ‘social norms’, defined as socially ascribed behavioral rules that are shared by individuals in a given society or group and which are followed because they are considered appropriate and normal behavior [30]. Some research uses personal attitudes interchangeably with a similar concept of personal normative beliefs, however for conceptual clarity, we interpret our measure as an attitude only [40].

3 In November 2012, North Kalimantan split from East Kalimantan becoming Indonesia’s 34th province.

4 The ARH data include four outliers; these respondents report their preferred marital age to be higher than 35 years (however mean preferred age remains similar at 25.5 years when we exclude these outliers). Similarly, when aforementioned four outliers are excluded from the multivariate analysis, the magnitude of the coefficients remains similar and the statistical significance of the results remains unchanged.

5 Although the indicator we operationalize is logged, we further investigate whether or not the significant relationship could be driven by the skewed distribution whereby there are some relatively richer females with a large number of siblings driving this relationship. Although significance level is diminished and no longer significant in marriage or cohabitation before 18 years and before 16 years, there is still a significant relationship with ever being married or cohabiting when we utilize dummy variables for higher than mean number of siblings (i.e. above four or five siblings). It could be that a better measure to operationalize would be number of female (instead of total) siblings, however as mentioned, overall this relationship warrants further investigation.

## References

[1] United Nations Children’s Fund (2014). Ending child marriage: progress and prospects.

[2] United Nations. Convention on the rights of the child. United Nations Treaty Series vol 1577. 1989. http://www.refworld.org/docid/3ae6b38f0.html. Accessed 7 Mar 2018.

[3] Gibbs CM, Wendt A, Peters S, Hogue CJ. The impact of early age at first childbirth on maternal and infant health. Paediatr Perinat Epidemiol. 2012; 10.1111/j.1365-3016.2012.01290.x. 10.1111/j.1365-3016.2012.01290.x PMC4562289 22742615

[4] World Health Organization. Health for the World’s Adolescents: A second chance in the second decade. 2014. http://apps.who.int/adolescent/second-decade/. Accessed 7 Mar 2018.

[5] Raj A, Boehmer U (2013). Girl child marriage and its association with national rates of HIV, maternal health, and infant mortality across 97 countries. Violence Against Women.

[6] Fall CH, Sachdev HS, Osmond C, Restrepo-Mendez MC, Victora C, Martorell R (2015). Association between maternal age at childbirth and child and adult outcomes in the offspring: a prospective study in five low-income and middle-income countries (COHORTS collaboration). Lancet Glob Health.

[7] Fall CH, Osmond C, Haazen DS, Sachdev HS, Victora C, Martorell R (2016). Disadvantages of having an adolescent mother. Lancet Glob Health.

[8] Brown G (2012). Out of wedlock, into school: combating child marriage through education.

[9] Peterman A, Bleck J, Palermo T (2015). Age and intimate partner violence: an analysis of global trends among women experiencing victimization in 30 developing countries. J Adolesc Health.

[10] Kidman R (2016). Child marriage and intimate partner violence: a comparative study of 34 countries. Int J Epidemiol.

[11] United Nations General Assembly. Transforming our world: the 2030 agenda for sustainable development (a/res/70/1). New York: United Nations; 2015.

[12] Temmerman M (2015). Research priorities to address violence against women and girls. Lancet.

[13] United Nations Department of Economic and Social Affairs. World fertility patterns. 2009. http://www.un.org/esa/population/publications/worldfertility2009/worldfertility2009.htm. Accessed 7 March 2018.

[14] Burn J, Evenhuis M (2014). Just married, just a child: child marriage in the indo-Pacific region.

[15] Malhotra A, Warner A, McGonagle A, Lee-Rife S (2011). Solutions to end child marriage.

[16] Kamal S, Hassan C (2015). Child marriage in Bangladesh: trends and determinants. J Biosoc Sci.

[17] Maswikwa B, Richter L, Kaufman J, Nandi A (2015). Minimum marriage age laws and the prevalence of child marriage and adolescent birth: evidence from sub-Saharan Africa. Int Perspect Sex Reprod Health.

[18] UNICEF. Hidden in plain sight: a statistical analysis of violence against children. New York: UNICEF; 2014.

[19] Statistics Indonesia (BPS), National Population and Family Planning Board, Ministry of Health (2013). ICF international. Indonesia demographic health survey.

[20] UNICEF. Violence against children in East Asia and the Pacific: a regional review and synthesis of findings. Bangkok: UNICEF; 2014.

[21] Statistics Indonesia (BPS), UNICEF. Child marriage in Indonesia: progress on pause. Jakarta: UNICEF; 2016.

[22] World Bank. World development indicators*.* 2015. data.worldbank.org/indicator/NY.GDP.MKTP.KD.ZG. Accessed 7 March 2018.

[23] UNICEF. Working toward progress with equity under decentralization: the situation of women and children in Indonesia 2000–2010. Jakarta: UNICEF; 2011.

[24] United Nations Economic and Social Council. Country Programme document: Indonesia. New York: United Nations Economic and Social Council; 2015.

[25] United Nations Committee on the Rights of the Child. Concluding observations on the combined third and fourth periodic reports of Indonesia. Geneva: United Nations; 2014.

[26] Coram Children’s Legal Centre. Legal protection from violence: analysis of domestic laws relating to violence against children in ASEAN states. Bangkok: UNICEF; 2015.Coram Children’s Legal Centre, Plan International. Getting the evidence: Asia child marriage initiative. London: Coram Children’s Legal Centre; 2015.

[27] United Nations Economic and Social Council. Report of the Inter-Agency and Expert Group on Sustainable Development Goal Indicators (E/CN/3/2016/2/Rev.2), Annex IV. 2016. http://unstats.un.org/unsd/statcom/47th-session/documents/2016-2-IAEG-SDGs-Rev1-E.pdf Accessed 7 March 2018.

[28] Statistics Indonesia (BPS), National Population and Family Planning Board, Kementerian Kesehatan, ICF International. Demographic and Health Survey 2012: Adolescent reproductive health. Jakarta: BPS; 2013.

[29] Neal SE, Hosegood V (2015). How reliable are reports of early adolescent reproductive and sexual health events in demographic and health surveys?. Int Perspect Sex Reprod Health.

[30] Cislaghi B, Heise L. Measuring gender-related social norms: report of a meeting, Baltimore Maryland, June 14 -15, 2016. Learning group on social norms and gender-based violence of the London School of Hygiene and Tropical Medicine; 2016.

[31] Wooldridge JM. Introductory econometrics: a modern approach (upper level economics titles). 5^th^ edition. Boston, MA: Cengage Learning; 2012.

[32] Palermo T, Peterman A (2009). Are female orphans at risk for early marriage, sexual debut and teen pregnancy? Evidence from sub-Saharan Africa. Stud Fam Plan.

[33] Peterman A, Schwab B, Roy S, Hidrobo M, Gilligan D (2015). Measuring women’s decisionmaking: indicator choice and survey design experiments from cash and food transfer evaluations in Ecuador, Uganda and Yemen (discussion paper #01453).

[34] Van den Bold M, Quisumbing A, Gillespie S (2013). Women’s empowerment and nutrition: an evidence review (discussion paper #01294).

[35] Marshan JN, Rakhmadi MF (2014). Rizky M.

[36] Mammen K, Paxson C (2000). Women's work and economic development. J Econ Perspect.

[37] Marinescu I, Triyana M (2016). The sources of wage growth in a developing country. IZA Journal of Labor & Development.

[38] Kalamar A, Lee-Rife S, Hindin M (2016). Interventions to prevent child marriage among young people in low- and middle-income countries: a systematic review of the published and gray literature. J Adolesc Health.

[39] Bruce J. Violence against adolescent girls: a fundamental challenge to meaningful equality. Popline. 2012. http://www.popline.org/node/572817. Accessed 7 March 2018. 7 Accessed 11 February 2017.

[40] Mackie G, Moneti F, Denny E, Shakya H. What are social norms? How are they measured. San Diego. University of California. 2012;

